# Dynamic changes in peripheral inflammation as a risk factor for perioperative sleep disturbances in elderly patients undergoing laparoscopic hepatobiliary surgery

**DOI:** 10.3389/fneur.2025.1537780

**Published:** 2025-04-16

**Authors:** Lai Wei, Xiaoyu Zhu, Yiming Zhao, Yi Zou, Tao Hu, Qian Huang, Jieqiong Li, Bingbing Pan, Gaoyin Kong, Siyou Tan, Wenyan Chen

**Affiliations:** ^1^Department of Anesthesiology, Hunan Provincial People’s Hospital (The First Affiliated Hospital of Hunan Normal University), Changsha, China; ^2^Perioperative Enhanced Recovery Anesthesia Clinical Medical Research Center of Hunan Province, Changsha, China; ^3^The ERAS Anesthesia Technology Innovation Center of Changsha, Changsha, China

**Keywords:** perioperative sleep disturbances, peripheral inflammation, surgery, immune cells, elderly

## Abstract

**Background:**

Elderly surgical patients are at high risk of perioperative sleep disturbances (PSD), and the underlying pathogenic mechanisms remain unclear. The relationship between peripheral inflammatory status and PSD pathogenesis currently lacks substantial clinical evidence.

**Objective:**

This study aims to evaluate the association between peripheral inflammation indicators and PSD in elderly patients undergoing laparoscopic hepatobiliary surgery, and to analyze the dynamic changes in peripheral inflammation in PSD patients throughout the perioperative period.

**Method and materials:**

Using retrospective data, this study compares peripheral inflammatory markers (NLR, MLR, PLR, SII, IL-6, and IL-10) in patients with PSD vs. those with normal sleep patterns before and after surgery. Receiver operating characteristic (ROC) curves were employed to evaluate the discriminative power of these indicators for PSD. Logistic regression models were employed to assess risk associations between inflammatory markers and PSD. Dynamic changes in peripheral inflammation were compared before surgery, on the day the surgery ended, and 1 day post-surgery between patients with PSD and those with normal sleep, exploring potential correlations with PSD pathogenesis.

**Result:**

The study ultimately included clinical data from 156 patients. Findings indicated that elevated NLR and SII levels before and after surgery, alongside decreased plasma IL-10 levels post-surgery, are associated with a higher incidence of PSD. Peripheral inflammatory markers on the day of surgery were not significantly predictive of post-PSD. Multivariable logistic regression analyses identified NLR, SII, IL-6, and IL-10 as independent predictors of pre-PSD, while NLR, SII, and IL-10 remained independently associated with post-PSD.

**Conclusion:**

Dynamic changes in peripheral inflammation during the perioperative period are associated with PSD in elderly patients undergoing laparoscopic hepatobiliary surgery. These findings may support the early identification and screening of high-risk PSD patients, providing new insights into the underlying mechanisms of PSD pathogenesis.

## 1 Introduction

The incidence of sleep disturbances increases with age, placing elderly surgical patients at particularly high risk for perioperative sleep disturbance (PSD) (1, 2). PSD has been linked to heightened perioperative pain sensitivity, reduced immunity, cardiovascular complications, postoperative delirium, and other severe outcomes (1, 3, 4). Clinically, PSD remains a dilemma due to insufficient attention from both healthcare providers and patients, overshadowed by the primary disease, and a limited understanding of PSD’s underlying pathogenesis (5). Given PSD’s association with serious clinical outcomes, including compromised patient recovery, addressing PSD preoperatively and managing postoperative sleep crises are crucial for promoting rapid recovery (6). Therefore, the prevention and treatment of PSD are urgent priorities, and further investigation into its pathogenic mechanisms could support early identification and targeted intervention.

The perioperative period is characterized by significant fluctuations in peripheral inflammation, and growing evidence suggests a link between peripheral inflammation and reciprocal modulation of cerebral neurological functions (7, 8). Sleep, a biologically conserved behavior across species, is regulated by the central nervous system; thus, impaired neurological function may present as abnormal sleep patterns (9–11). Current understanding of both the correlation between peripheral inflammation and PSD in elderly surgical patients, as well as the perioperative dynamic changes in peripheral inflammation among elderly PSD patients, remains relatively limited. To investigate the correlation between peripheral inflammation and PSD in elderly surgical patients, this study collected data on perioperative peripheral inflammation and sleep assessments at our institution. We compared peripheral inflammation levels between patients with PSD and those with normal sleep both preoperatively and postoperatively, analyzing the relationship between PSD and inflammatory status. Given that factors like surgical site and perioperative pain status can influence PSD and peripheral inflammation, we focused on patients undergoing laparoscopic hepatobiliary surgery, applying strict inclusion and exclusion criteria to control for confounding effects. Sleep data were collected using the Numerical Rating Scale for Sleep (NRS-S) and the Athens Insomnia Scale (AIS), both reliable tools for perioperative sleep assessment (12–14). Recent clinical evidence has established blood cell component ratios as sensitive markers of inflammation and stress, such as the neutrophil-to-lymphocyte ratio (NLR) and the systemic inflammation index (SII) (15). In this study, we assessed peripheral inflammation based on routine blood test results, calculating NLR, platelet-to-lymphocyte ratio (PLR), monocyte-to-lymphocyte ratio (MLR), and SII, and measuring plasma interleukins (IL-6 and IL-10). This study aims to clarify the relationship between peripheral inflammation and PSD pathogenesis in elderly surgical patients, identifying peripheral blood cell types and molecules potentially involved in PSD pathogenesis. These findings are expected to enhance the understanding of PSD prevention and support strategies for improved postoperative recovery.

## 2 Methods and materials

### 2.1 Study population

Data for this study were retrospectively collected from patients undergoing laparoscopic hepatobiliary surgery at the First Hospital Affiliated to Hunan Normal University (Hunan Provincial People’s Hospital) between December 1, 2022, and December 30, 2023, who had undergone perioperative sleep assessments. Ethical approval was obtained from the Ethics Committee of the First Hospital Affiliated to Hunan Normal University (Approval No. [2022]-114), granting permission to access medical records. Inclusion criteria were patients aged 65–90 years, classified as ASA I–III, and scheduled for elective laparoscopic hepatobiliary surgery. Exclusion criteria included the presence of hematologic disease; end-stage conditions such as severe organ failure; history or postoperative diagnosis of malignancy; severe psychiatric illness; chronic inflammatory disease; infections; history of substance abuse; BMI ≥ 30; ongoing antibacterial or antiviral therapy; hypersensitivity; decompensated cardiac, pulmonary, or cerebrovascular disease; blood transfusions; medications potentially affecting routine blood test results; incomplete data; obstructive sleep apnea syndrome (OSAS); history of epilepsy, thyroid disease, food or drug allergies, chronic pain, or Parkinson’s disease; surgeries ending after 6:00 p.m.; and pre- or postoperative NRS-pain (NRS-P) scores ≥ 3, as well as postoperative neurocognitive deficits. Patients diagnosed with PSD were assigned to the patient group, while those with normal sleep assessments were assigned to the control group.

For each patient, demographic data (age, gender, and body mass index (BMI)), comorbidities, history of smoking and alcohol use, disease severity or physical status (NYHA classification and ASA classification), perioperative indices (operation duration, recovery time, anesthetic agents, and blood loss), pain status (NRS-P), peripheral inflammation markers (NLR, MLR, PLR, SII, IL-6, and IL-10), and PSD status (NRS-S and AIS) were collected.

### 2.2 PSD diagnosis

All participants included in the study completed the AIS and NRS-S scales to assess sleep status preoperatively and 1 day postoperatively. The AIS score is a commonly used and recognized valid tool for sleep assessment. The scale consists of 8 questions, each of which is assigned a score based on severity, and has a total score of 24, with higher scores representing poorer sleep. The NRS-S score ranges from 0 to 10, with 0 indicating excellent or good sleep and 10 representing an inability to sleep throughout the night. PSD was defined as an NRS-S score ≥ 6 or an NRS-S score ≥ 6.

### 2.3 Data for peripheral inflammation

Peripheral blood cell counts were retrospectively obtained from patient medical records at three time points: preoperatively, on the day the surgery was completed (POD0), and on postoperative day 1 (POD1). These counts were measured using a clinical blood cell analyzer (XE-2100, Sysmex, Japan) and included absolute counts of neutrophils (NEU), lymphocytes (LYM), monocytes (MON), and platelets (PLT). Various inflammatory indices were then calculated as follows: (1) NLR as NEU/LYM; (2) PLR as PLT/LYM; (3) MLR as MON/LYM; and (4) SII as PLT × NEU/LYM. Additionally, we collected data on peripheral inflammatory factors, including IL-6 and IL-10.

### 2.4 Statistical analysis

Statistical analysis was performed with SPSS, version 27.0 (Statistical Package for Social Sciences). The normal distribution of the variables was examined using the Kolmogorov–Smirnov test. Continuous data were presented as mean (SD) and compared using the unpaired or paired t-test, or one-way ANOVA if distributed normally. Data that were not normally distributed were reported as median (IQR) and analyzed using the Mann–Whitney test. Categorical variables were reported as number (%) and compared using χ^2^ or Fisher exact test, as appropriate. Correlation analysis was performed by Spearman’s correlation analysis. Receiver operating characteristic (ROC) curves were plotted separately for each index of peripheral inflammation, and the diagnostic efficacy was assessed by comparing the area under the curve (AUC). Binary logistic regression models were implemented to evaluate associations between peripheral inflammatory marker levels and PSD risk, with PSD status as the dependent variable and inflammatory biomarkers as independent variables. Subsequent analyses further adjusted for covariate interactions between biomarkers and age, gender, preoperative AIS and NRS-S scores, and recovery length. All association estimates were quantified as odds ratios (ORs) with corresponding 95% confidence intervals (CIs). Two-sided *p* < 0.05 was considered to be statistically significant.

## 3 Results

### 3.1 Patients’ characteristics

**Figure 1:**
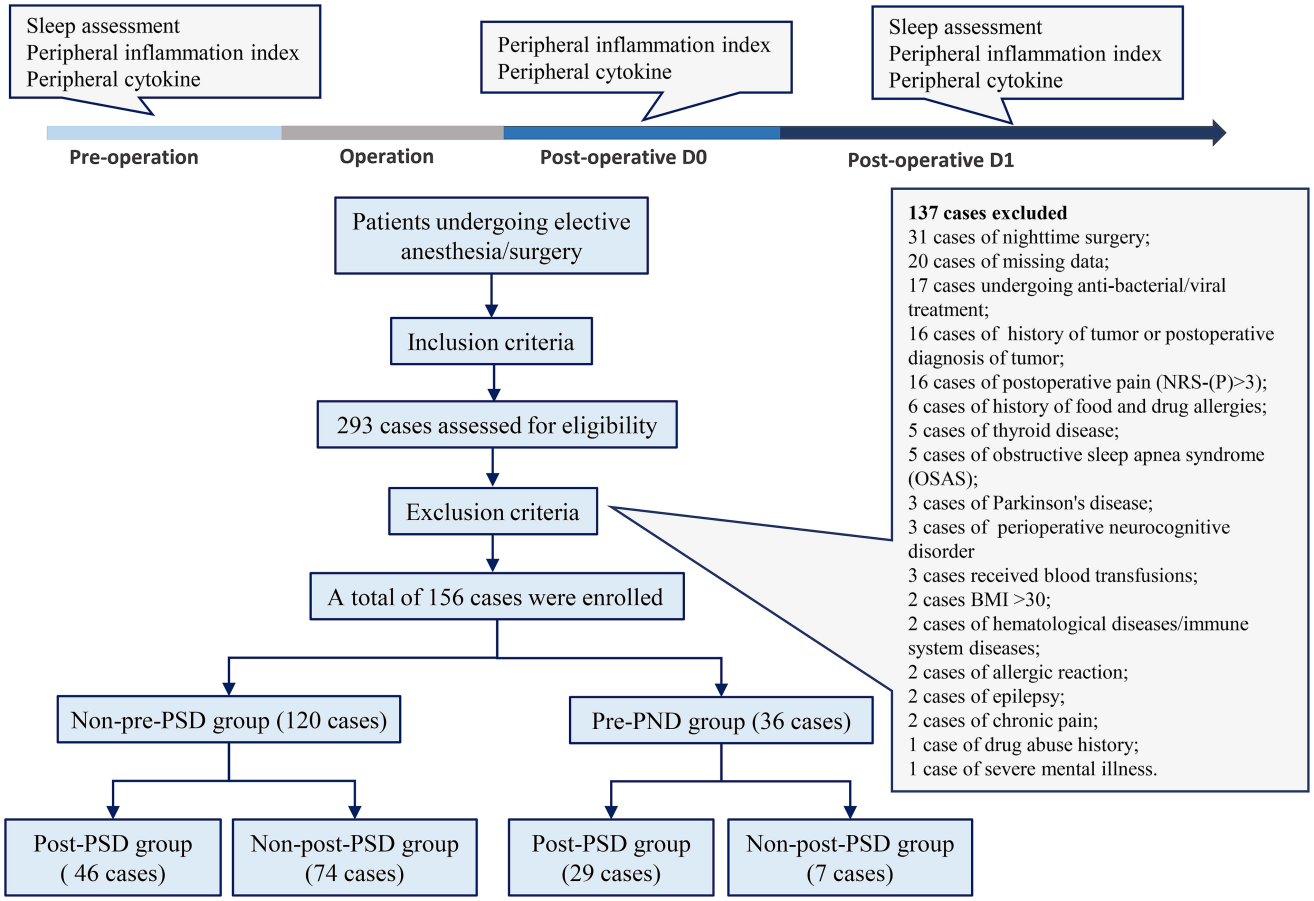
Participant flowchart.

A total of 293 patients were assessed for eligibility, with 137 patients excluded based on the exclusion criteria (Figure 1). A final sample of 156 patients was enrolled in the study, including 81 (52%) males and 75 (48%) females, with a mean age of 72.5 years. Two cohorts were created to identify risk factors related to peripheral inflammation that may influence PSD before and after surgery. In Cohort I (Table 1), patients were divided into those with preoperative PSD (pre-PSD; *n* = 36, 23%) and those without pre-PSD (*n* = 120, 77%). In Cohort II (Table 2), patients were divided based on postoperative PSD status, with 75 (48%) in the post-PSD group and 81 (52%) in the non-post-PSD group. We compared age, gender, BMI, smoking status, alcohol use, coronary heart disease, hypertension, diabetes mellitus, chronic obstructive pulmonary disease (COPD), thyroid disease, and physical condition between the groups to ensure comparability. The post-PSD and non-post-PSD groups differed in recovery time as well as preoperative AIS and NRS-S scores (Table 2).

**Table 1:** Comparison of baseline characteristics between the non-pre-PSD and the pre-PSD groups.

Baseline data	Non-pre-PSD (*n* = 120)	pre-PSD (*n* = 36)	*p*-value
Age (y), median (IQR)	72 (68–77)	71 (66–75)	0.46
Gender (male), No. (%)	67 (56)	18 (50)	0.57
BMI, median (IQR)	18.5 (16.4–20.3)	19 (16.7–20.2)	0.19
ASA classification			
I, No. (%)	25 (21)	6 (17)	0.85
II, No. (%)	71 (59)	22 (61)	
III, No. (%)	24 (20)	8 (22)	
Hypertension, No. (%)	32 (27)	13 (36)	0.30
Diabetes mellitus, No. (%)	8 (7)	5 (14)	0.18
Coronary heart disease, No. (%)	13 (11)	6 (17)	0.39
COPD, No. (%)	7 (6)	3 (8)	0.70
NYHA classification			
I, No. (%)	98 (82)	25 (69)	0.16
II, No. (%)	22 (18)	11 (31)	
Drinking, No. (%)	28 (23)	7 (19)	0.82
Smoking, No. (%)	29 (24)	10 (28)	0.67
Pre-NRS-P, median (IQR)	1 (0–1)	1 (0–1)	0.23
Pre-AIS, median (IQR)	3 (3–4)	6 (6–7)	<0.001
Pre-NRS-S, median (IQR)	3 (2–4)	6 (5–6)	<0.001

AIS, Athens Insomnia Scale; ASA, American Society of Anesthesiologists; BMI, body mass index; COPD, chronic obstructive pulmonary disease; NRS-P, numeric rating scale-pain; NRS-S, numeric rating scale-sleep; NYHA, New York Heart Association; PSD, perioperative sleep disturbance. Data presented as median (IQR) were compared using the Mann–Whitney test. Data reported as the number of patients (%) were compared using the χ^2^ test or Fisher exact test.

**Table 2:** Comparison of baseline characteristics between the non-post-PSD and the post-PSD groups.

Baseline data	Non-post-PSD (*n* = 81)	Post-PSD (*n* = 75)	*p*-value
Age (y), median (IQR)	72 (66–76)	72 (68–77)	0.60
Gender (male), No. (%)	45 (56)	40 (53)	0.87
BMI, median (IQR)	19.5 (16.9–22.9)	18.3 (16.3–21.5)	0.08
ASA classification			
I, No. (%)	17 (21)	14 (19)	0.80
II, No. (%)	49 (60)	44 (58)	
III, No. (%)	15 (19)	17 (23)	
Hypertension, No. (%)	25 (31)	20 (27)	0.60
Diabetes mellitus, No. (%)	5 (6)	8 (11)	0.39
Coronary heart disease, No. (%)	11 (14)	8 (11)	0.81
COPD, No. (%)	6 (7)	4 (5)	0.75
NYHA classification			
I, No. (%)	65 (80)	58 (77)	0.70
II, No. (%)	16 (20)	17 (23)	
Drinking, No. (%)	16 (20)	19 (25)	0.45
Smoking, No. (%)	22 (27)	17 (23)	0.58
Preoperative variables			
Pre-NRS-P, median (IQR)	0 (0–1)	1 (0–1)	0.27
Pre-PSD, No. (%)	7 (8.6)	29 (38.7)	<0.001
Pre-AIS, median (IQR)	3 (3–4)	3 (3–6)	0.007
Pre-NRS-S, median (IQR)	3 (2–4)	4 (2–6)	0.03
Intraoperative variables			
Operation duration (min), median (IQR)	90 (70–120)	105 (85–125)	0.13
Recovery length (min), median (IQR)	22 (18–30)	26 (21–32)	0.02
Bleeding (mL), median (IQR)	20 (20–50)	20 (2–100)	0.88
Porpofol (mg/kg), median (IQR)	3.69 (2.83, 4.75)	4.05 (3.22, 4.84)	0.14
Sufentanil (ug/kg), mean (SD)	0.67 (0.20)	0.71 (0.20)	0.14
Remifentanil (ug/kg), median (IQR)	7.64 (5.56, 10.38)	7.30 (5.52, 8.67)	0.17
Vecuronium bromide (mg/kg), median (IQR)	0.19 (0.14, 0.24)	0.18 (0.15, 0.21)	0.73
Postoperative variables			
Analgesic remedy, No. (%)	14 (17)	12 (16)	0.99
Pod0-NRS-P, median (IQR)	2 (2–2)	2 (2–2)	0.12
Pod1-NRS-P, median (IQR)	1 (1–2)	1 (1–2)	0.22
Post-AIS, median (IQR)	5 (4–5)	9 (7–14)	<0.001
Post-NRS-S, median (IQR)	5 (4–5)	7 (6–8)	<0.001

AIS, Athens Insomnia Scale; ASA, American Society of Anesthesiologists; BMI, body mass index; COPD, chronic obstructive pulmonary disease; NRS-P, numeric rating scale-pain; NRS-S, numeric rating scale-sleep; NYHA, New York Heart Association; PSD, perioperative sleep disturbance. Data presented as median (IQR) were compared using the Mann–Whitney test. Data reported as the number of patients (%) were compared using the χ^2^ test or Fisher exact test.

### 3.2 High levels of preoperative peripheral inflammation are associated with the development of both pre-/post-PSD

**Figure 2:**
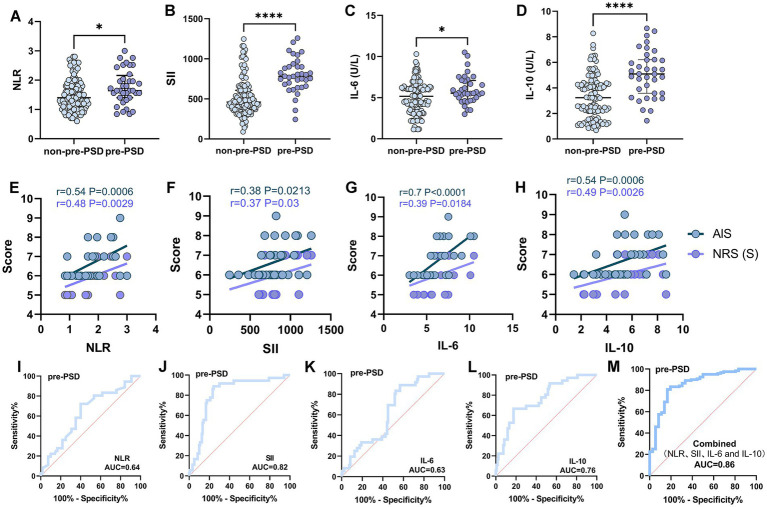
Comparison and correlation analysis of peripheral inflammatory parameters in patients with non-pre-PSD and pre-PSD. **(A–D)** Comparison of peripheral blood NLR **(A)**, SII **(B)**, IL-6 **(C)**, and IL-10 **(D)** levels between non-pre-PSD and pre-PSD patients; **(E–H)** Correlation of preoperative NLR **(E)**, SII **(F)**, IL-6 **(G)**, and IL-10 **(H)** levels with sleep status in pre-PSD patients (*n* = 36); **(I–L)** ROC curves illustrating the discriminatory efficacy of preoperative NLR **(I)**, SII **(J)**, IL-6 **(K)**, and IL-10 **(L)** between pre-PSD and non-pre-PSD groups; **(M)** Evaluation of the discriminatory ability of combined preoperative NLR, SII, IL-6, and IL-10 on the pre-PSD and non-pre-PSD patients. ^*^p < 0.05; ^****^p < 0.0001.

We initially analyzed preoperative sleep quality and peripheral inflammation in elderly patients undergoing elective laparoscopic hepatobiliary surgery. Our findings indicated that levels of peripheral inflammatory markers, including NLR, SII, IL-6, and IL-10, were significantly higher in patients with pre-PSD compared to those with normal sleep patterns (non-pre-PSD) (Figures 2A–D). Correlation analysis further revealed significant linear relationships between preoperative NLR, SII, IL-6, and IL-10 levels and preoperative sleep scores assessed by the AIS and NRS-S (Figures 2E–H). Results of the unadjusted analyses and multivariable models are presented in Table 3. Models were created to adjust the associations between the biomarkers and outcomes for age and gender based on previous studies as deemed clinically relevant (1, 16). After adjustment, the preoperative level of NLR, SII, IL-6, and IL-10 were all associated with pre-PSD in separate models. Subsequently, we constructed ROC curves to evaluate the diagnostic efficacy of these markers for pre-PSD. Among these, preoperative SII showed the highest diagnostic value (AUC = 0.82), followed by NLR, IL-6, and IL-10, with AUCs of 0.64, 0.63, and 0.76, respectively (Figures 2I–L). The combined diagnostic efficacy of all four markers achieved an 0.86 (Figure 2M). These findings suggest a significant association between preoperative peripheral inflammatory status and sleep disturbances.

**Table 3:** Preoperative NLR, SII, IL-6, and IL-10 were associated with the risk of pre-PSD.

	Presence of pre-PSD			
Items	Unadjusted		Adjusted for age and sex	
	OR (95% CI)	*p*-value	OR (95% CI)	*p*-value
NLR	2.18 (1.15, 4.21)	0.018	2.17 (1.13, 4.25)	0.022
SII	1.04 (1.03, 1.06)	<0.001	1.05 (1.03, 1.07)	<0.001
IL-6	1.29 (1.07, 1.60)	0.012	1.30 (1.07, 1.60)	0.011
IL-10	1.76 (1.40, 2.28)	<0.001	1.80 (1.42, 2.34)	<0.001

OR, odds ratio; 95% CI, 95% confidence interval.

**Figure 3:**
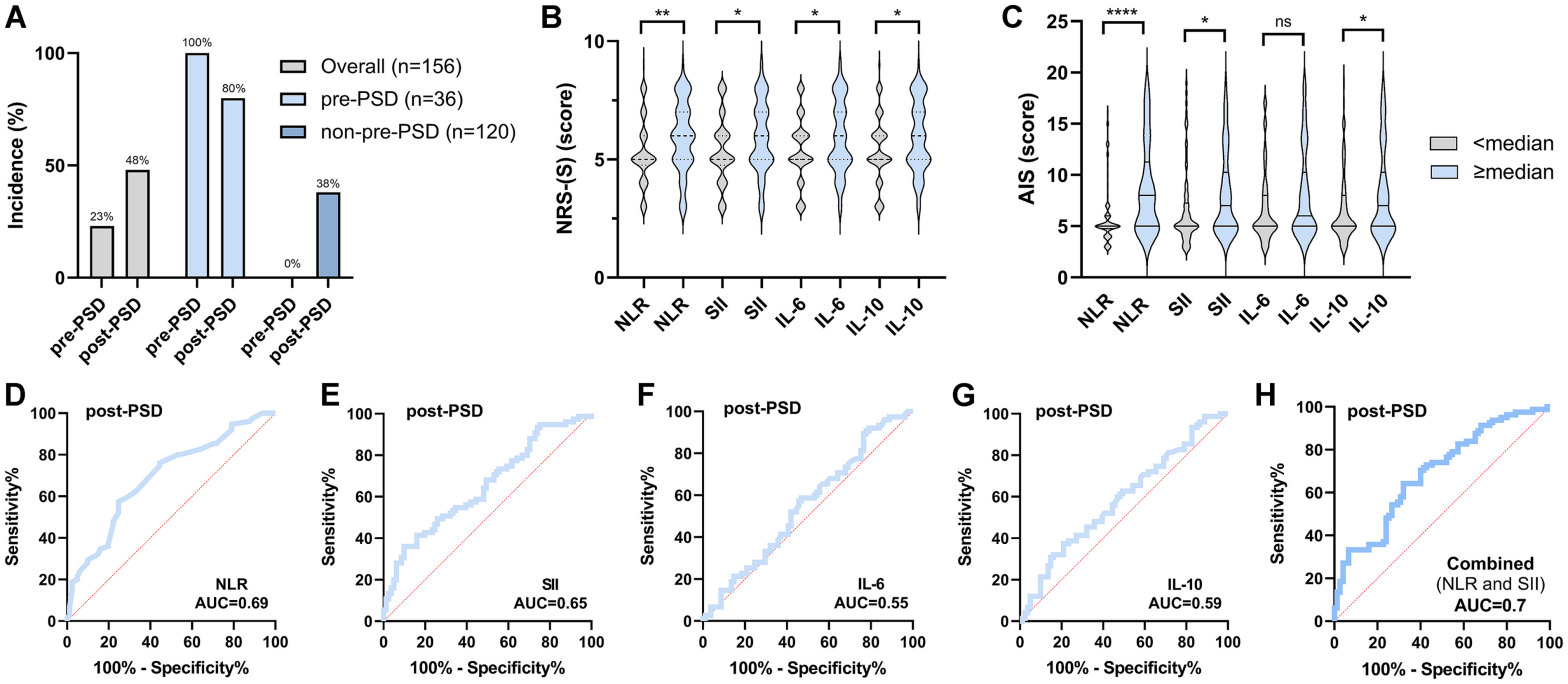
Preoperative sleep condition and peripheral inflammatory status significantly affect postoperative sleep. **(A)** Comparison of the incidence of post-PSD between patients in the pre-PSD and non-pre-PSD groups; **(B)** Effect of preoperative high/low levels of NLR, SII, IL-6, and IL-10 on postoperative NRS-S scores; **(C)** Effect of preoperative high/low levels of NLR, SII, IL-6, and IL-10 on postoperative AIS scores; **(D–G)** ROC curves of preoperative peripheral blood NLR **(D)**, SII **(E)**, IL-6 **(F)**, and IL-10 **(G)** differentiating between post-PSD and non-post-PSD patients; **(H)** Evaluation of the discriminatory ability of combined preoperative NLR, SII, IL-6, and IL-10 on the post-PSD and non-post-PSD patients. ns, not significant; ^*^p < 0.05; ^**^p < 0.01; ^****^p < 0.0001.

Since the incidence of post-PSD was significantly higher in patients with pre-PSD than in those with normal preoperative sleep (80% in the pre-PSD group vs. 38% in the non-pre-PSD group, *p* < 0.0001), this suggests that postoperative sleep is substantially influenced by preoperative sleep conditions (Figure 3A). To further examine this relationship, we divided preoperative peripheral inflammation levels into two categories, using the median as a cutoff, and assessed their effects on postoperative sleep. The results indicated that postoperative sleep scores were higher (reflecting poorer sleep) in patients with elevated preoperative levels (≥ median) of NLR, SII, IL-6, and IL-10 (Figures 3B,C). Additionally, the AUC values for preoperative NLR, SII, IL-6, and IL-10 in predicting post-PSD were 0.69, 0.65, 0.55, and 0.59, respectively, with a combined diagnostic/predictive AUC of 0.7 (Figures 3D–H).

The differences in peripheral MLR and PLR between patients with pre-PSD and those with normal sleep were not statistically significant, with AUC values for pre- and post-PSD ranging from 0.51 to 0.58 (Supplementary Figure S1). Given that the inflammatory index is derived from blood cell components, we compared levels of NEU, LYM, MON, and PLT between the pre-PSD and non-pre-PSD groups. The results indicated that, aside from NEU levels, there were no significant differences in these blood cell components between the two groups (Supplementary Figure S1).

### 3.3 Peripheral inflammatory features at POD0 have no significant predictive potential for post-PSD

Only elevated levels (≥ median) of peripheral IL-6 on POD0 were associated with significant differences in postoperative AIS scores (Supplementary Figure S2). The AUC values for the predictive efficacy of NLR, SII, MLR, PLR, IL-6, and IL-10 in discriminating post-PSD were 0.55, 0.52, 0.53, 0.54, 0.56, and 0.52, respectively (Supplementary Figure S2). These findings suggest that peripheral inflammation on POD0 has limited discriminatory ability for post-PSD.

### 3.4 Post-PSD patients are accompanied by persistent activation of peripheral inflammation

**Figure 4:**
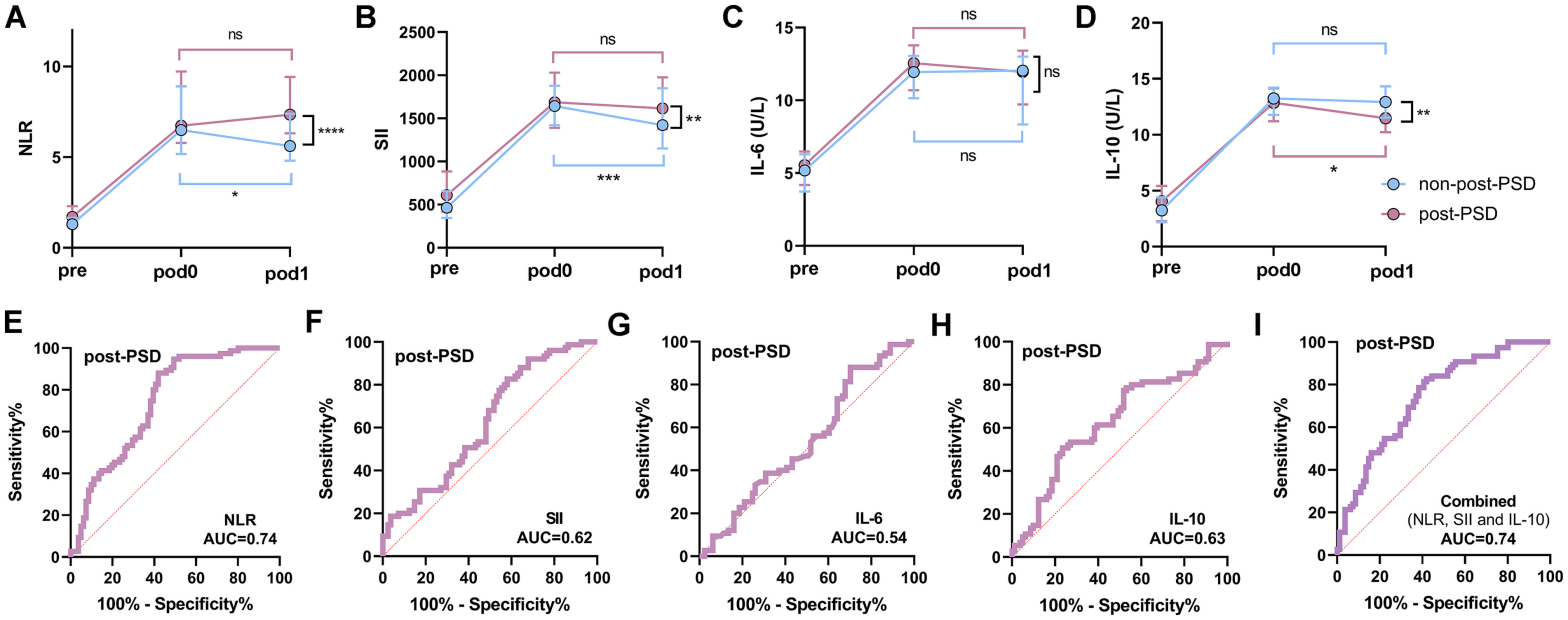
Dynamic analysis of perioperative peripheral inflammatory status in post-PSD and non-post-PSD patients. **(A–D)** Comparison of peripheral blood NLR **(A)**, SII **(B)**, IL-6 **(C)**, and IL-10 **(D)** levels between post-PSD and non-post-PSD patients at different periods; **(E–H)** ROC curves of postoperative (POD1) peripheral blood NLR **(E)**, SII **(F)**, IL-6 **(G)**, and IL-10 **(H)** differentiating between post-PSD and non-post-PSD patients; **(I)** Evaluation of the discriminatory ability of combined postoperative NLR, SII, IL-6, and IL-10 on the post-PSD and non-post-PSD patients. ns, not significant; ^*^p < 0.05; ^**^p < 0.01; ^***^p < 0.001; ^****^p < 0.0001.

This study included 81 elderly surgical patients with normal postoperative sleep (non-post-PSD) and 75 patients with post-PSD. Peripheral inflammation data for these patients were collected preoperatively, as well as on POD0 and POD1, to observe dynamic changes in inflammation associated with the development of PSD. Compared to the non-post-PSD group, post-PSD patients exhibited significantly higher NLR and SII levels and lower IL-10 levels at POD1, with no significant difference observed in IL-6 levels between the two groups (Figures 4A–D). In the crude models, increased postoperative level of NLR and SII, lower level of IL-10, were associated with an increase incidence of post-PSD. This association persisted after adjustment for age, gender, pre-AIS, pre-NRS-S, and recovery time (Table 4). The AUC values for postoperative NLR, SII, IL-6, and IL-10 in predicting post-PSD were 0.74, 0.62, 0.54, and 0.63, respectively (Figures 4E–H), with a combined AUC of 0.74 for all four indices (Figure 4I). No significant differences were found in MLR or PLR levels between the two groups on POD1 (Supplementary Figure S3).

**Table 4:** Postoperative NLR, SII, and IL-10 were associated with the risk of pre-PSD.

	Presence of post-PSD			
Items	Unadjusted		Adjusted for age, sex, pre-AIS, pre-NRS-S, and recovery length	
	OR (95% CI)	*p*-value	OR (95% CI)	*p*-value
NLR	1.23 (1.08, 1.44)	0.007	1.23 (1.07, 1.45)	0.01
SII	1.01 (1.00, 1.02)	0.005	1.01 (1.00, 1.02)	0.009
IL-6	1.08 (0.97, 1.21)	0.17	1.04 (0.92, 1.17)	0.55
IL-10	0.84 (0.72, 0.96)	0.02	0.83 (0.71, 0.95)	0.01

OR, odds ratio; 95% CI, 95% confidence interval.

A significant elevation in inflammatory markers was observed in all patients on POD0 compared to the preoperative period. Analysis of the non-post-PSD group revealed a substantial decrease in NLR and SII levels at POD1 compared to POD0, while IL-6 and IL-10 levels remained largely unchanged (Figures 4A–D). In contrast, in post-PSD patients, NLR, SII, and IL-6 levels at POD1 showed no significant difference from POD0, while IL-10 levels were significantly lower at POD1 (Figures 4A–D). These findings indicate distinct patterns of peripheral inflammatory changes between the two groups in the postoperative period, with post-PSD patients experiencing sustained peripheral inflammation from POD0 to POD1, suggesting an imbalance in the body’s proinflammatory and anti-inflammatory responses.

Given that NLR and SII are calculated based on NEU, LYM, and PLT counts, we further analyzed these blood cell levels in both groups. The results showed that NEU levels were significantly higher in post-PSD patients than in non-post-PSD patients at POD1, while LYM levels were significantly lower, with no significant differences observed in PLT or MON levels between the two groups (Supplementary Figure S4). In both non-post-PSD and post-PSD groups, NEU and LYM levels at POD1 were not significantly different from POD0 (Supplementary Figure S5). Interestingly, we found that surgery/anesthesia resulted in a significant increase in peripheral NEU levels and a significant decrease in LYM counts in both groups (Supplementary Figure S5). These findings suggest that the dynamic changes in NEU and LYM levels induced by surgery and anesthesia contribute to the alterations in NLR and SII and may be associated with the development of post-PSD.

## 4 Discussion

This study provides evidence of a correlation between peripheral inflammatory status and PSD in elderly surgical patients. Our findings revealed that elevated preoperative peripheral blood levels of NLR, SII, IL-6, and IL-10 were associated with increased pre-PSD incidence, and post-PSD occurrence correlated with higher NLR/SII and lower IL-10 levels. Specifically, those with poorer preoperative sleep showed significantly heightened peripheral inflammatory activation. Additionally, the results suggest that preoperative sleep disturbances are more likely to persist into the postoperative period. Based on these findings, we stratified patients by preoperative inflammatory marker levels to assess the effects of high vs. low preoperative peripheral inflammation on postoperative sleep outcomes. Patients with elevated preoperative peripheral inflammation demonstrated poorer postoperative sleep scores, suggesting that perioperative sleep status in elderly surgical patients is influenced by preoperative or long-term sleep conditions, potentially mediated by peripheral inflammation.

We also found that while peripheral inflammation levels at POD0 had little discriminatory power for postoperative PSD, all patients experienced a significant inflammatory response likely due to anesthesia- and surgery-induced stress and tissue injury. During the perioperative period, patients are exposed to a complex array of stressors, which may obscure the relationship between peripheral inflammation and PSD. Among elderly patients who developed PSD postoperatively, peripheral inflammation levels were elevated relative to those without PSD, although IL-6 levels did not differ significantly. Notably, postoperative PSD patients had lower peripheral IL-10 levels, which is interesting as higher preoperative IL-10 levels were positively associated with PSD pathogenesis. In the postoperative phase, IL-10 levels were significantly elevated in all patients compared with preoperative levels. Given IL-10’s role as an anti-inflammatory mediator, contributing to the initiation of anti-inflammatory responses and the maintenance of immune-inflammatory homeostasis (17). These findings may reflect the body’s attempt to restore balance in preoperative PSD patients. Importantly, postoperative PSD patients had relatively lower IL-10 levels than non-PSD patients, suggesting a reduced anti-inflammatory capacity that could exacerbate inflammation, potentially contributing to PSD pathogenesis.

In this study, NLR and SII were screened as significant factors associated with PSD due to remarkable level differences. Higher levels of both were associated with the occurrence of PSD both preoperatively and at POD1. Both NLR and SII reflect the peripheral systemic inflammatory immune state of the body, and several studies have provided evidence of the value of NLR and SII for the diagnosis/prognosis of related diseases. For example, NLR predicts in-hospital mortality/survival in patients with extensive burns after hospital admission and in patients with sepsis (18, 19). Similarly, in the general population, SII is significantly associated with all-cause cardiovascular disease causation (20). To clarify the reasons leading to elevated levels of NLR and SII, we analyzed the differences in preoperative and postoperative NEU and LYM, as well as PLT levels in different cohorts of patients. We found that peripheral NEU levels were significantly higher in older patients with preoperative sleep disturbances than in patients with normal sleep, whereas LYM and PLT levels were similar in both groups, so higher NLR and SII values may be associated with higher NEU levels. Notably, in contrast to the significant increase in NEU after surgery compared with a preoperative period, peripheral LYM levels were reduced in both groups at POD0 and POD1. Active neutrophil proliferation *in vivo* due to surgical and tissue trauma has been demonstrated in several studies (21, 22), and there is also evidence of functional inhibition and reduction in the number of peripheral LYMs induced by anesthesia/surgical exposure, as well as accelerated programmed death processes (23–27), which is in line with the reduction in the number of LYMs observed in this study.

The mechanisms underlying the association between peripheral inflammatory alterations and perioperative sleep homeostasis in elderly populations represent a critically important issue, with three key aspects warranting in-depth exploration: First, can fluctuations in peripheral inflammation affect sleep? Second, can sleep disturbances induce peripheral inflammatory activation? Third, are elderly individuals more susceptible to sleep dysregulation under peripheral inflammatory states? Experimental animal studies have indeed identified bidirectional sleep-immune interactions (28–30). On the one hand, inflammatory cytokines exhibit circadian rhythmicity, and their daytime elevation during illness or irregular sleep patterns disrupt sleep architecture (28). Previous research has shown that rodents exposed to lipopolysaccharide (LPS)-induced inflammation display altered sleep architecture, including dysregulation in the ratio of REM to non-REM sleep, increased EEG delta activity in non-REM sleep, and impaired sleep continuity (31–34). Additionally, sleep deprivation in mice is linked to compromised immune function, resulting in increased susceptibility to infectious and inflammatory diseases (35). Prolonged sleep deprivation in mammals has been associated with the accumulation of circulating neutrophils and a cytokine storm-like syndrome (36, 37). These findings suggest a bidirectional relationship between peripheral inflammation and sleep status. In elderly patients, age-related immunosenescence (characterized by elevated baseline inflammation and impaired anti-inflammatory capacity) (38) and compromised BBB integrity (39, 40) create synergistic vulnerabilities. Peripherally activated inflammatory responses can disrupt functional brain network connectivity, while excessive peripheral inflammation may induce neuroinflammation. Pro-inflammatory mediators released by activated glial cells can lead to synaptic dysfunction, accelerated programmed neuronal death, and a range of neurocognitive complications (39, 41). These findings collectively suggest that elderly surgical patients may not only exhibit increased susceptibility to sleep disturbances due to compromised peripheral inflammatory homeostasis but also experience exacerbated systemic immune-inflammatory responses secondary to abnormal sleep patterns, thereby establishing a self-reinforcing pathophysiological loop.

There are limitations to this study. We retrospectively collected perioperative data from elderly patients undergoing hepatobiliary surgery. Whereas both sleep and peripheral immune status are influenced by a variety of factors, we tried to control for effects by excluding factors such as pain, Parkinson’s disease, and chronic inflammation, but as an observational study, the ability to infer causality is limited. Retrospective study designs introduce the risk of bias and unmeasured sources of confounding by other factors that cannot be fully mitigated by the use of exclusion strategies. We stratified the level of peripheral inflammation preoperatively and postoperatively to explore the sleep of people with different levels of peripheral inflammation, respectively. This analysis was considered exploratory because we did not compare baseline data for the stratified populations, and the sample size for stratification was determined based on the population of participants already included in this study, which did not ensure adequate test efficacy. Other limitations are that the sample size of this study was small, partly because we had limited access to cases with documented sleep assessments. In addition, this study did not include the inclusion of sleep architecture data. Our subsequent research agenda will focus on conducting prospective studies with expanded sample sizes and broader inclusion of peripheral inflammatory biomarkers, coupled with prolonged observation windows and integration of multidimensional sleep assessment methodologies (including polysomnography and actigraphy), to systematically investigate the impact of peripheral inflammation on specific sleep architecture features such as sleep duration, arousal frequency, REM sleep, and NREM sleep characteristics.

## 5 Conclusion

This retrospective study investigated the correlation between postoperative sleep disturbances (PSD) and perioperative peripheral inflammatory dynamics in elderly patients undergoing laparoscopic hepatobiliary surgery. Key findings revealed that elevated preoperative/postoperative NLR and SII, coupled with reduced postoperative IL-10 levels, were significantly associated with increased PSD incidence. These discoveries provide novel insights for early identification of high-risk PSD populations and establish new pathways for elucidating the pathogenic mechanisms underlying PSD development.

## Data Availability

The original contributions presented in the study are included in the article/Supplementary material, further inquiries can be directed to the corresponding authors.
