# Supplementary material for: Dynamic changes in peripheral inflammation as a risk factor for perioperative sleep disturbances in elderly patients undergoing laparoscopic hepatobiliary surgery
Source: Front Neurol. 2025 Apr 16;16:1537780. doi: 10.3389/fneur.2025.1537780 (PMC12040671; doi:10.3389/fneur.2025.1537780)
Supplement: Supplementary file 1 [file Supplementary_file_1.DOCX]

**Supplementary materials**


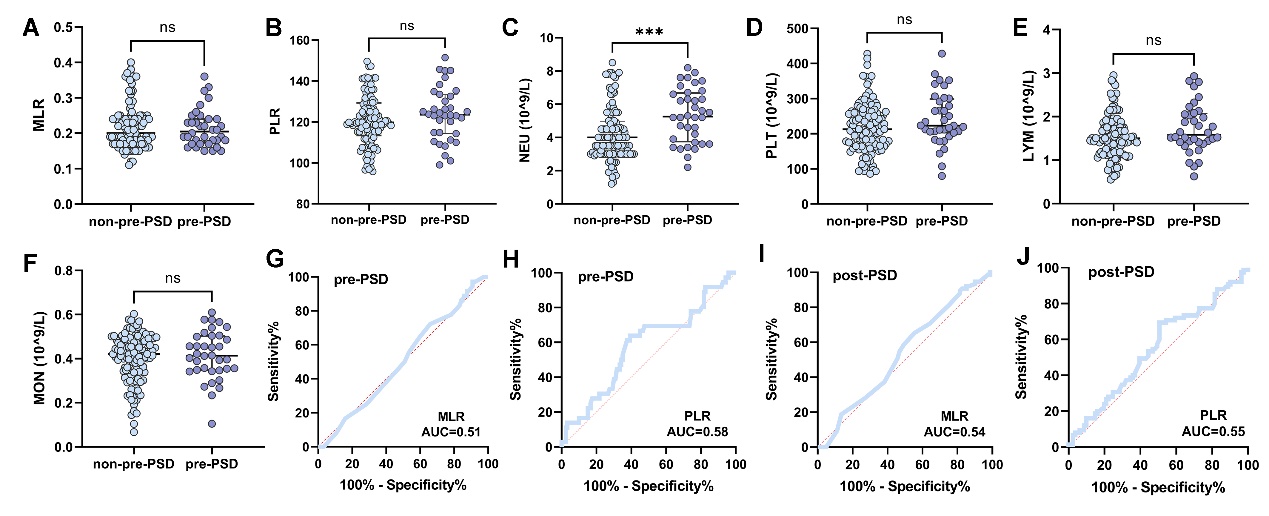


**Figure S1 Comparison of peripheral inflammatory parameters in patients with non-pre-PSD and pre-PSD. (A-F)** Comparison of peripheral blood MLR (A), PLR (B), NEU (C), PLT (D), LYM (E), and MON (F) levels between non-pre-PSD and pre-PSD patients; **(G-H)** ROC curves of preoperative peripheral blood MLR (G) and PLR (H) differentiating between pre-PSD and non-pre-PSD patients; **(I-J)** ROC curves of preoperative peripheral blood MLR (G) and PLR (H) differentiating between post-PSD and non-post-PSD patients. ns, not significant; ***, *P*<0.001.


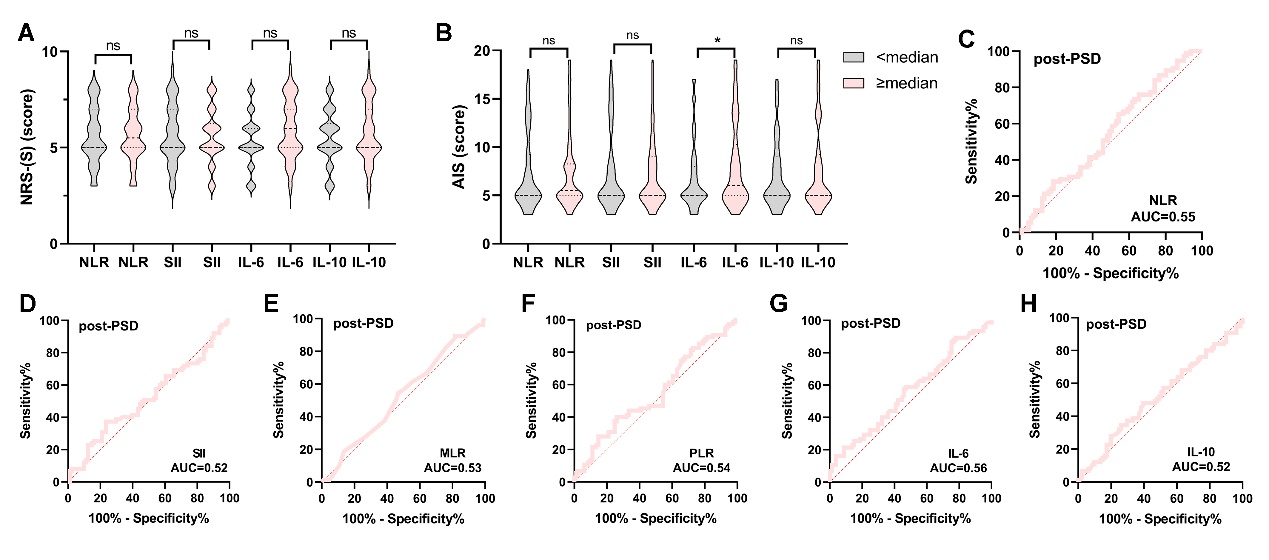


**Figure S2 Peripheral inflammatory status at POD0 has no significant discriminatory ability for post-PSD.** **(A)** Effect of postoperative (POD0) high/low levels of NLR, SII, IL-6, and IL-10 on postoperative NRS-S scores; **(B)** Effect of postoperative (POD0) high/low levels of NLR, SII, IL-6, and IL-10 on postoperative AIS scores; **(C-H)** ROC curves of postoperative (POD0) peripheral blood NLR (C), SII (D), MLR (E), PLR (F), IL-6 (G) and IL-10 (H) differentiating between post-PSD and non-post-PSD patients. ns, not significant; *, *P*<0.05.


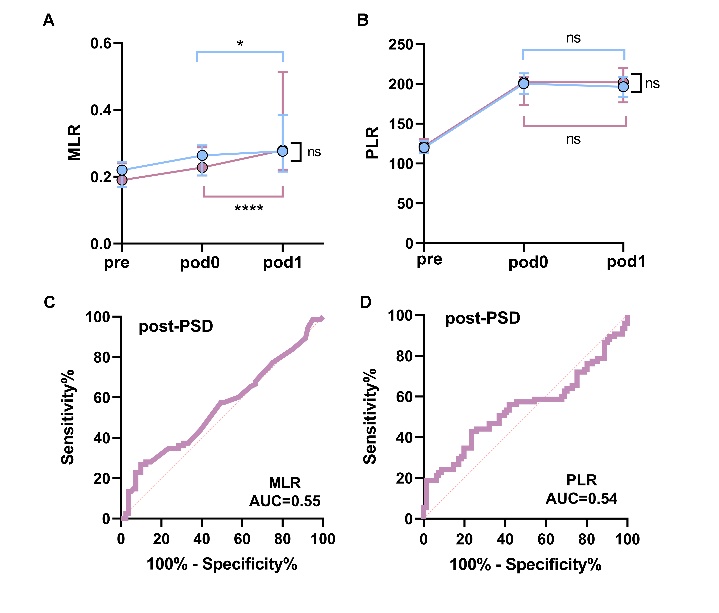


**Figure S3 Dynamic analysis of perioperative peripheral inflammatory status in post-PSD and non-post-PSD patients.** **(A-B)** Comparison of peripheral blood MLR(A) and PLR (B) levels between post-PSD and non-post-PSD patients at different periods; **(C-D)** ROC curves of postoperative (POD1) peripheral blood MLR (C) and PLR (D) differentiating between post-PSD and non-post-PSD patients. ns, not significant; ****, *P*<0.0001.


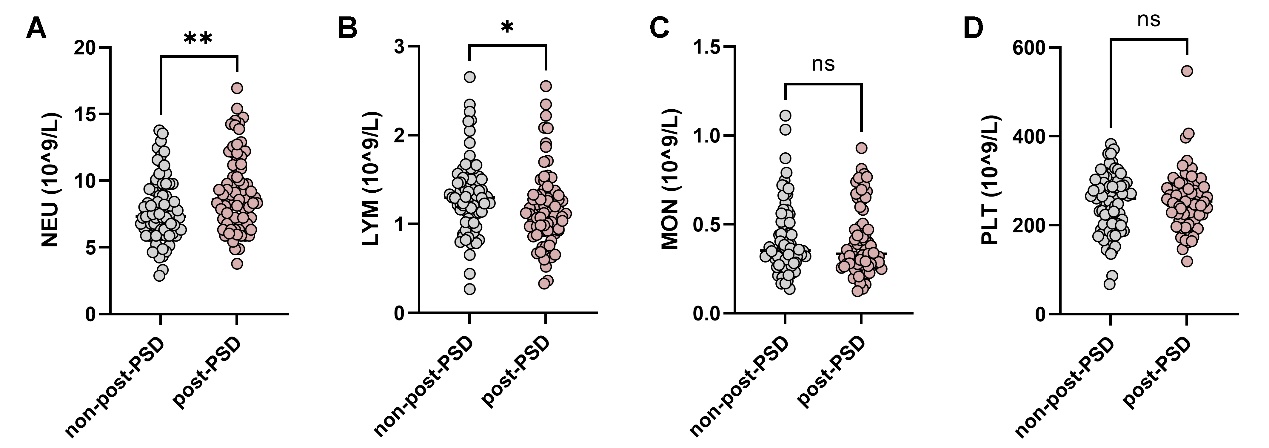


**Figure S4 Comparison of peripheral inflammatory parameters in patients with non-post-PSD and post-PSD. (A-D)** Comparison of peripheral blood NEU (A), LYM (B), MON (C), and PLT (D) levels between non-post-PSD and post-PSD patients. *, *P*<0.05; **, *P*<0.01.


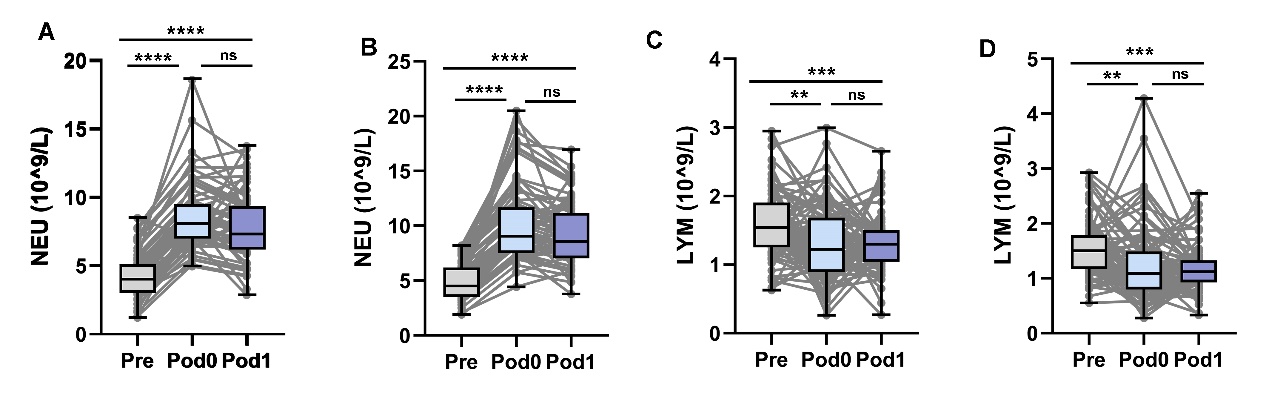


**Figure S5 Dynamic changes in peripheral blood NEU and LYM counts in post-PSD and non-post-PSD patients.** **(A)** Comparison of peripheral blood NEU counts in non-post-PSD patients at preoperative periods, POD0, and POD1; **(B)** Comparison of peripheral blood NEU counts in post-PSD patients at preoperative periods, POD0, and POD1; **(C)** Comparison of peripheral blood LYM counts in non-post-PSD patients at preoperative periods, POD0 and POD1; **(D)** Comparison of peripheral blood LYM counts in post-PSD patients at preoperative periods, POD0 and POD1.ns, not significant; **, *P*<0.01; ***, *P*<0.001; ****, *P*<0.0001.
